# Evaluation and Management of Pyogenic Spondylodiscitis: A Review

**DOI:** 10.3390/jcm14103477

**Published:** 2025-05-15

**Authors:** Rick Placide, Julie Reznicek

**Affiliations:** 1 Department of Orthopedic Surgery, VCU Health, Medical College of Virginia, Richmond, VA 23298-0153, USA; ricky.placide@vcuhealth.org; 2 Division of Infectious Diseases, VCU Health, Medical College of Virginia, Richmond, VA 23298-0153, USA

**Keywords:** spondylodiscitis, vertebral osteomyelitis, hematogenous, acute phase reactants, spine surgery, spine infection

## Abstract

Spondylodiscitis is a devastating invasive infection that can lead to debilitating pain, motor weakness, or paralysis, even with appropriate medical and surgical treatment. Over the past two decades, there has been a worldwide increase in the incidence of spondylodiscitis, which can be attributed to a higher prevalence of various risk factors including intravenous drug use, hemodialysis, and spinal surgeries. The lumbar spine is the most likely region to be affected, with *Staphylococcus aureus* being the predominant pathogen. Management of spondylodiscitis requires a multi-disciplinary approach, with close coordination between the spinal surgeon and the infectious diseases specialist. Clinicians should become familiar with the epidemiology and presentation of patients with suspected spondylodiscitis because timely diagnosis and treatment may lead to improved outcomes. This unique review incorporates the perspectives from infectious disease and spine surgery specialists.

## 1. Introduction

Pyogenic spondylodiscitis (PSD) is a bacterial infection of the spinal column. It can also be referred to as osteodiscitis, vertebral osteomyelitis, discitis, or spondylodiscitis. Although the terminology may imply the involvement of specific structures, this infection is essentially a progression of the same disease, and we will be using these terms interchangeably throughout this manuscript. These infections can occur de novo or spontaneously and are referred to as native vertebral osteomyelitis (NVO) or after surgical interventions (post-operative vertebral osteomyelitis, PVO). PSD is a condition with the potential for severe morbidity and mortality, and it is not uncommon for a patient with PSD to have a protracted course of signs and symptoms before the correct diagnosis is made [1]. This delay in diagnosis has been associated with poorer outcomes [1].

## 2. Pathophysiology

There are three main pathways that can lead to spondylodiscitis, the most common being hematogenous spread via paired segmental arteries that terminate in the vascular vertebral endplate. Typically, two adjacent vertebrae are infected, with eventual spread to the intervening avascular disc. In children, these arterial anastomoses extend into the disc, making the disc the initial site of infection. Compared with the posterior elements, the increased vascularity and cellular marrow of the vertebral body make it an optimal environment for bacterial seeding [2].

Potential pathogens can enter the blood stream through various means including open wounds, minor skin trauma, via the oral cavity, or the urinary or gastrointestinal tracts. Identification of the pathogen can often help the clinician decipher the origin of the infection [3].

Spondylodiscitis can also develop secondary to direct pathogen inoculation during surgery, procedures (e.g., lumbar puncture), or trauma. The third mechanism of action is contiguous spread from a neighboring structure such as a ruptured esophagus or diverticular abscess.

*Staphylococcus aureus* (methicillin sensitive and methicillin resistant) remains the most commonly isolated organism in vertebral osteomyelitis, regardless of whether or not spinal instrumentation is involved. More indolent organisms, such as coagulase-negative staphylococci and *Cutibacterium acnes*, typically only cause infection in the setting of indwelling hardware, except in the following unique situation. Patients who are on hemodialysis can develop native vertebral osteomyelitis from *Staphylococcus epidermidis*, likely because of repeated vascular access, which can lead to recurrent transient bacteremia. For example, the relative risk of bacteremia with a tunneled cuffed central venous catheter is 8.49 and 1.47 for arteriovenous grafts [3]. Non-pyogenic spine infections are typically seen in the immunocompromised population or in patients who are at risk due to travel or exposure. The most common non-pyogenic pathogens include *Mycobacterium tuberculosis*, atypical Mycobacteria, fungi, or *Brucella* species.

## 3. Epidemiology/Etiology

Native vertebral osteomyelitis (NVO) accounts for 3–5% of all cases of osteomyelitis, and in patients over the age of 50, it is the most common manifestation of hematogenous osteomyelitis [4]. Over the past three decades, the incidence has been increasing, likely secondary to our aging population, the increased number of immunocompromised patients, increased use of intravascular devices, and improved diagnostic testing [5]. The male-to-female ratio remains at approximately 1.5.

Risk factors for the development of spondylodiscitis are similar to those seen in other severe infections and can be broadly categorized into underlying illnesses or medications that impair the immune system (e.g., diabetes mellitus, steroids, advanced liver disease) or conditions that increase the risk of bacteremia such as intravenous drug use or the presence of indwelling catheters [6].

A recent large epidemiological study of over 9000 patients confirmed the anatomic distribution outlined in previous studies. The lumbar spine continues to be the most common location for spondylodiscitis (56.2%), followed by the thoracic spine (18.3%), and cervical spine (6.7%) [7]. Infection involving the thoracic spine is most likely to result in a neurologic deficit, while epidural abscesses are more commonly seen in the cervical spine. Spinal tuberculosis classically affects the thoracic spine, specifically the thoracolumbar junction, with the vertebral body itself being primarily involved, resulting in this particular spine infection often being labeled as “disc sparing”.

## 4. History/Physical Exam

The most common presenting symptom in patients with PSD is an insidious onset of back pain (~90% of patients) [8]. The lumbar spine has the highest incidence of PSD, followed by the thoracic spine, then the cervical spine [9]. A smaller percentage of patients may present with additional constitutional signs and symptoms such as fevers, chills, malaise (~65%), and even fewer patients present with a neurological deficit (5–30%) [8]. Since most cases of PSD are a result of hematogenous spread from another source (skin infection, urinary tract infection, endocarditis), a thorough history and physical exam are crucial to avoid missing the diagnosis of PSD when evaluating a patient with back or neck pain.

Although back pain is a common complaint, there are elements of the history and exam that can help include PSD in the differential diagnosis. The pain is typically insidious and can be present for several months before the patient seeks care. However, the pain can be abrupt and severe, especially when associated with an epidural abscess or vertebral body pathological fracture. History should include recent travel, invasive procedures, and any other recent infections [6]. Some conditions that increase the risk of PSD include older age, diabetes, immunocompromised states, end-stage renal disease, liver disease, alcoholism, obesity, recent invasive spine procedures, and intravenous drug abuse.

In addition to the basic vital signs, a musculoskeletal and neurological exam is essential. The patient may appear in distress and unable to find a comfortable position. In the region of the patients’ pain, the paraspinal muscles may be tender to palpate and be in spasm. Dermatome and myotome examination of the upper extremities, trunk, and lower extremities may reveal a sensory or motor deficit. When coupled with an exam of the deep tendon reflexes and pathological reflexes, a spinal level of the lesion may be identified. Examination of the heart, lungs, abdomen, and lymph nodes may help identify the source of the infection.

## 5. Initial Work-Up

All patients who present with back pain and concern for spondylodiscitis should have a detailed motor and sensory examination performed, in addition to the following laboratory studies: two sets of bacterial blood cultures obtained from two different peripheral sites prior to the initiation of antibiotics, erythrocyte sedimentation rate (ESR) [1], C-reactive protein (CRP), complete blood count (CBC), basic metabolic profile (BMP), and liver function tests (LFTs). In select patients with risk factors for less common causes of spondylodiscitis, additional testing is warranted, which may include fungal blood cultures, *Brucella* serologies, endemic fungal antigen/antibody testing [10], and tuberculosis testing via an interferon-gamma release assay (IGRA). If the patient has a history of injecting drugs, HIV and hepatitis testing should also be performed.

Each of these diagnostic results must be viewed as only one data point in a conglomerate of information, and the diagnosis of spondylodiscitis should never be made or rejected based on just one of them. Patients often present without leukocytosis, despite radiographic evidence of infection and spine destruction, and some patients may have persistently elevated inflammatory markers secondary to other causes of chronic inflammation (e.g., uncontrolled diabetes mellitus) [2].

If blood cultures or serologies fail to establish a microbiologic diagnosis, image guided aspiration is indicated [10], however, the sensitivity of this diagnostic modality is quite low, often below 50% [11,12]. Contributing factors have been postulated, including the site of biopsy, needle gauge, and number of core samples, but a definitive impact of any of these has not been established [13]. Intuitively, one would think that any antibiotic exposure would affect the biopsy results, but similar to what is seen in other areas in the orthopedic literature [14,15], there does not seem to be a negative impact of a few doses of antimicrobials on the diagnostic yield of the biopsy. However, the effect of pre-biopsy antimicrobials is likely cumulative, and any unnecessary antibiotics should be withheld if possible [16]. The current Infectious Diseases Society Guidelines recommend repeat biopsy if the initial biopsy is nondiagnostic [10], but this is not always a feasible option. Biopsy samples should be sent for bacterial culture as well as histopathological examination, which can identify acute or chronic inflammation, granulomas, or possible malignancy. Using next generation sequencing (NGS) on biopsy or surgical samples has also been investigated. One retrospective study found that when using histopathology results as the reference, NGS had increased sensitivity compared with the traditional culture (70.3% vs. 14.8%), but significantly lower specificity (75% vs. 100%), likely because of skin flora contamination; this is a common cause of false positives when DNA-based diagnostic methods are utilized [17].

## 6. Imaging

**Figure 1:**
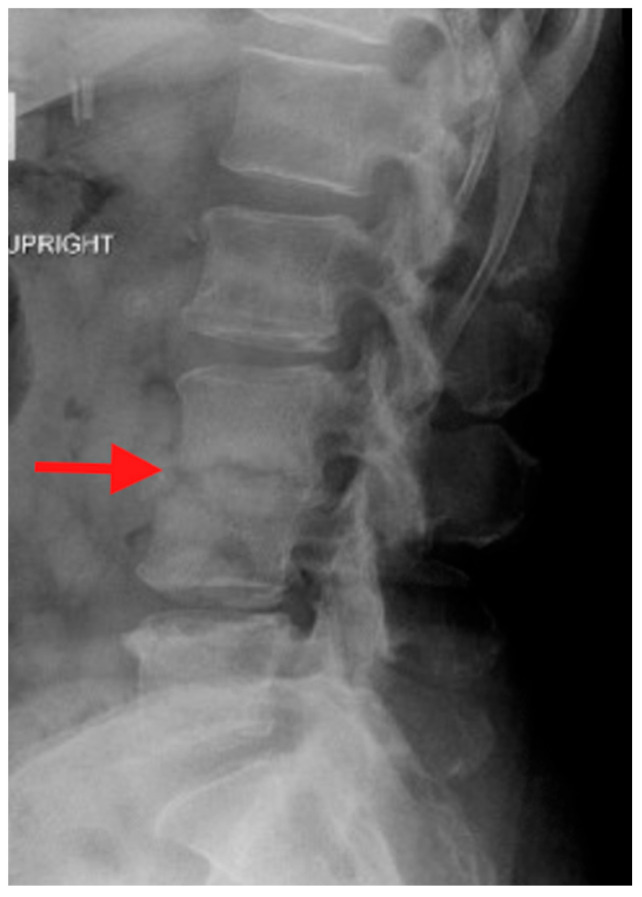
Upright lateral plain radiograph of the lumbar spine. The red arrow is pointing to the L3–4 disc space. Irregularity of the vertebral endplates and collapse into a kyphotic deformity can be seen.

Plain radiographs are typically the first imaging study obtained when evaluating a patient with back or neck pain. In the case of PSD, plain radiographs may be normal, particularly early in the course of the infection. Eventual findings include disc space narrowing and bony resorption near the endplates. However, plain radiographs are important as they are easy to obtain in an upright position and during various motions such as flexion and extension of the spine. With these attributes, plain radiographs can demonstrate spinal deformity and instability associated with PSD (Figure 1).

**Figure 2:**
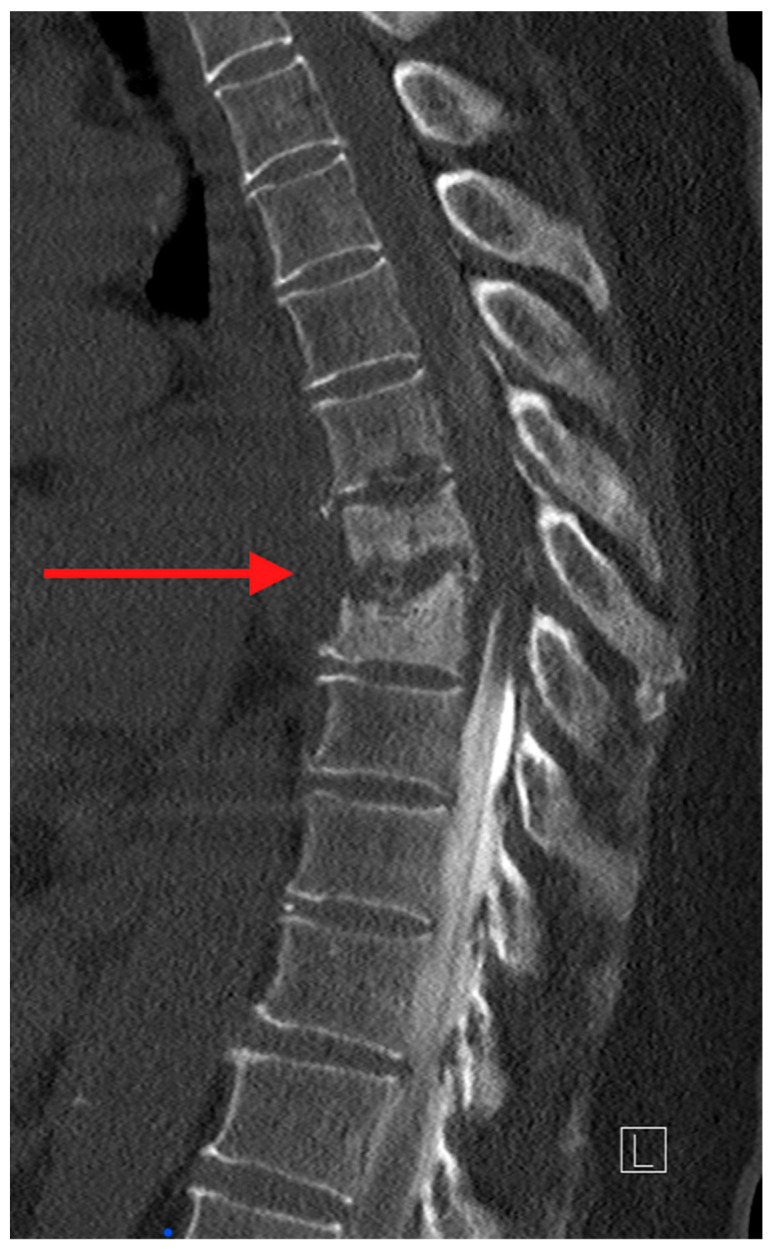
Sagittal CT myelogram of the thoracic spine. The red arrow is pointing to the destructive changes of several vertebral bodies in the mid-thoracic spine. Also noted is the myelogram dye in the spinal canal. It was injected caudal to cranial and the abrupt disappearance of the column of dye at the infected level suggests high-grade cord compression [4].

Computed tomography (CT) is the ideal imaging study to evaluate bony detail. CT allows for an accurate assessment of bony destruction, identifying subtle pathological fractures and assisting in evaluating spinal stability/instability (Figure 2). In the case where surgery is being considered, the ability to obtain 3-dimensional image reconstruction makes CT critical for surgical planning.

**Figure 3:**
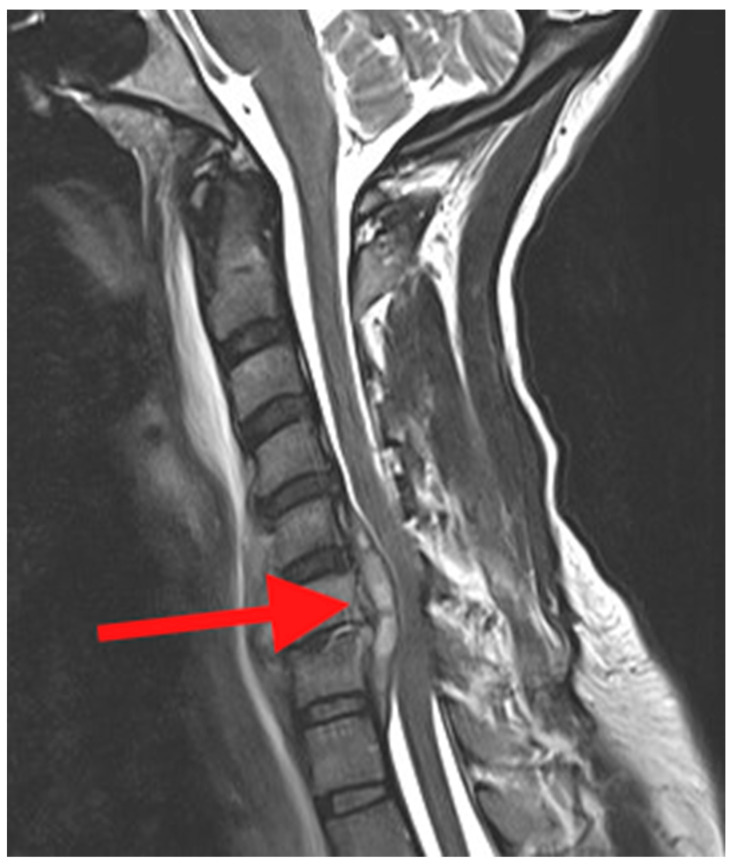
Sagittal T2 weighted MRI of the cervical spine. The red arrow points to an epidural abscess behind the C5, 6, and 7 vertebral bodies and compression of the ventral spinal cord.

Magnetic resonance imaging (MRI) is the imaging modality of choice in the evaluation of PSD. Compared with CT, MRI would demonstrate a better visualization of the soft tissues including any associated abscesses, muscles and ligaments, spinal cord, cauda equina, and nerve roots (Figure 3). MRI will also provide earlier detection of the infection compared with plain radiographs and CT. It is recommended if PSD is identified in one region of the spine, and that the other regions be imaged to identify “skip” lesions [18].

Fluorodeoxyglucose positron emission tomography (FDG-PET) can be an excellent diagnostic imaging modality, especially when MRI is not possible due to an implant that is not compatible with MRI. FDG-PET can differentiate between degenerative changes and PSD. However, neoplastic disease can also have an increased uptake of FDG, thereby making it difficult to differentiate between infection and tumor based solely on this imaging modality [19].

## 7. Treatment Decision Making

In patients with PSD, management is typically interdisciplinary and includes at least a general medical specialist, an infectious diseases specialist, and a spinal surgeon. Even when surgery is not part of the initial plan, a spinal surgery consult should be obtained to ensure timely outpatient follow-up. Ideally, an organism is identified via blood cultures or CT guided biopsy to provide targeted antibiotics. If cultures fail to identify a causative pathogen, patients are often discharged on a broad-spectrum antimicrobial regimen, which may include 2–3 medications. This does increase the risk for adverse effects and noncompliance. Occasionally, a spinal orthosis can help with pain control, but there are no data to suggest a brace effects the long-term outcome. Activity modification is typically recommended. Risk factors for the failure of conservative (non-operative) treatment are shown in Table 1 [20,21].

Indications for operative intervention in the patient with PSD include the need for an open biopsy, spinal instability/deformity, neurological compromise, intractable pain, and failed non-operative treatment. Spinal instability/deformity and neurological signs and symptoms include those that are present at initial evaluation or those that progress over time. In patients who have risk factors for failure of conservative treatment, earlier surgery has led to improved outcomes. Published grading schemes that categorize patients with SPD can help guide treatment as non-operative versus operative [22].

## 8. Antibiotic Management

The optimal duration and route of antimicrobial therapy for the treatment of spondylodiscitis continues to be debated, and more recent literature has shown the pendulum shifting toward shorter courses of intravenous antibiotics with more emphasis placed on oral options. The mantric recommendation of “6 weeks of intravenous antibiotics” for bone and joint infections dates to a landmark New England Journal article from 1970 [23], where the authors concluded that “osteomyelitis is rarely controlled without combination of careful, complete debridement and prolonged (4–6 weeks) of parenteral antibiotic therapy at high dosage”. This statement was likely defended by the limited bioavailability of the oral antibiotics that were available at that time. Over the past couple of decades, some have attempted to rebuke the belief that intravenous therapy is superior to oral therapy, but it was not until the large (>1000 patients), multi-center, randomized controlled, noninferiority OVIVA (Oral versus Intravenous Antibiotics for Bone and Joint infection) trial came onto the landscape in 2019 that the possibility of successful treatment with oral antibiotics could become the mainstay [24]. Many large medical centers in the United States have now created institutional guidelines for the treatment of bone and joint infections with oral antibiotics. This has in turn spawned much-needed manuscripts and will continue to do so. One recent group completed a retrospective review by comparing a pre and post guideline cohort and observed similar outcomes, specifically 90-day treatment failure [25]. One hundred and eighty-six patients were included in this study, and those patients discharged on oral antibiotics showed no difference in 90-day treatment failure when compared with patients discharged on intravenous antibiotics. A systemic review of twenty randomized controlled trials also supported the use of oral antibiotics in blood and bone infection including vertebral osteomyelitis and discitis [26].

Mirroring the change we are seeing with the recommended route of therapy, the duration of antibiotic therapy for spondylodiscitis has also evolved. Previous approaches to antimicrobial therapy suggested the extension of antimicrobials past the 6–8-week duration if the inflammatory markers had not decreased appropriately and/or the patient continued to have pain. In 2015, a landmark randomized controlled trial was published in the Lancet, which showed that in the majority of cases, a 6-week regimen was non-inferior to 12 weeks. Older patients (>75 y/o), patients with *Staphylococcus aureus* infections, and those with endocarditis or neurologic signs may benefit from a longer treatment course [11]. In our practice, we have found that if the patient continues to have debilitating pain after an appropriate antibiotic course, they will likely need spine stabilization and additional antibiotics are futile.

Patients who require stabilization surgery (e.g., anterior and/or posterior fusion) after an appropriate 6–8-week course of antimicrobials typically do not need an additional prolonged parenteral course. This suggestion is supported by a 3-year retrospective review involving 102 patients with spondylodiscitis who underwent a surgical intervention after receiving parenteral antimicrobials [27]. The authors had two major aims: identify independent risk factors for recurrence rates in this subset of patient who underwent surgery and compare the recurrence rates when short-term antibiotics (≤3 weeks) were prescribed post-operatively vs. a >3-week course. The two independent risk factors for recurrence were positive blood cultures and paraspinal abscess, and these patients were then classified as “high-risk”. These high-risk patients would likely benefit from an additional prolonged intravenous antimicrobial course (>3 weeks), while low-risk patients could receive a shorter course of parenteral antibiotics. Both groups of patients would then be transitioned to oral antibiotic suppression therapy, which is typically continued for 3–6 months, or possibly longer, depending on the maturation of the fusion, which is typically guided by plain films. Oral antibiotic suppression therapy is used in many different scenarios, usually involving retained prosthetic material that cannot be feasibly removed at that time. The optimal duration remains unknown, and providers must always weigh the risk of long-term antibiotics against the potential benefits [28].

## 9. Operative Management

When surgery is decided upon, the goals include decompression of the neural structures, adequate debridement of infected material, and stabilization. When there is compression of the spinal cord, cauda equina, or nerve roots, decompression is warranted. Compression of the neural elements may cause neurological deficits from the associated abscess, phlegmon, infected intervertebral disc, spinal deformity/instability, and bony fragments in the setting of a pathological fracture due to the infection. In addition to the compression of the neural structures, the infection is associated with local vasculitis, which creates a relative ischemia to the neural structures, worsening the neurological deficits.

The adequate debridement of infected material is critical for the outcome of eradication of the infection. There are a variety of techniques and instruments for debriding spinal infections, and debriding as much as is safely possible is indicated. Multiple specimens (three to four) for culture should be obtained at this time. Debridement needs to be aggressive enough to restore blood flow to the area, thus allowing antibiotics to reach the infection.

Surgical stabilization of the spine is an important part of the overall treatment. In the past, it was thought that placing implants at the time of debridement would lead to the implants becoming infected. While that is possible, it does not typically occur. The literature tells us that placing spinal hardware at the time of debriding an active infection does not increase the chance of recurrent infection or the need for hardware removal [29,30]. If decompression and debridement destabilize the spine, as it often does, adding stability is necessary to help prevent deformity progression, recurrent neural element compression, and infection eradication.

## 10. Follow-Up

It is essential that all patients discharged on intravenous antimicrobials have weekly labs performed to monitor for any adverse effects such as but not limited to acute kidney injury, drug induced hepatitis, leukopenia, or thrombocytopenia. The utility of weekly inflammatory markers (ESR,CRP) is unknown, but most groups agree that they should not be obtained until at least 4 weeks of antimicrobial therapy, given that they will remain falsely elevated secondary to surgery, prolonged hospitalization, or the initiation of intravenous antibiotics [10]. These laboratory values should also be evaluated in tandem with the patient’s clinical progress because in many cases, the patients will continue to improve clinically despite persistently elevated inflammatory markers [31].

In our practice, we are able to see this subset of patients at the same visit, so we can address their medical and surgical management congruently. This has proven to be very beneficial for the patients, but also for the clinicians involved. Patients are typically seen 2–3 weeks post-operatively, then at 6 weeks, and then every 3 months for the next 12–18 months. It must be highlighted here that even if patients are initially only treated medically, they should still be followed by a spinal surgeon in case motor or sensory deficits develop, which would require timely surgical intervention.

Patients should have a plain film at each follow-up visit for the first year to evaluate the hardware and fusion status. Routine follow-up MRI or CT is not indicated, unless the patient presents with new neurologic dysfunction or a change in their pain intensity or location because abnormalities seen on the initial MRI or CT typically show progression despite clinical improvement [32]. This lack of specificity parallels the limited utility of inflammatory markers in the post-operative period.

All patients should receive education about the reasonable expectations for pain levels and functional status. It often takes up to one year for patients to regain their full strength and endurance, and pain management may be indicated. Patients may also experience an impaired quality of life for many years following their initial diagnosis [33].

## 11. Conclusions

This review of pyogenic spondylodiscitis provides a comprehensive review of the latest literature and also provides a unique perspective from two clinicians who take care of this particular disease state in a multi-disciplinary clinic. Given the increasing incidence of both de novo and post-surgical spine infections, practitioners must always include infection on their differential diagnosis for a patient who presents with severe back pain. Despite a heightened awareness and sophisticated imaging, this diagnosis is often delayed, which directly correlates with poorer outcomes including paralysis. Patients benefit from a multi-disciplinary approach that should include a spinal surgeon, an infectious diseases specialist, the patients’ primary care physician, and a rehabilitation team. Further research is still needed to help identify which patients will clinically improve with medical management alone, and which ones would benefit from earlier surgical intervention.

## Figures and Tables

**Table 1:** Risk factors for the failure of conservative (non-operative) treatment in a patient with pyogenic spondylodiscitis.

Infection with methicillin-resistant ***Staphylococcus aureus***
Bacteremia
Associated epidural abscess
Osteomyelitis at another site
Neurological deficit/cord compression
Spinal instability
Diabetes mellitus
Age > 65
Intravenous drug use
Immune compromise
Cervical or thoracic spine involvement
Involvement of multiple spine levels
